# Quality of life in patients of corrosive esophageal stricture treated with endoscopic dilatation

**DOI:** 10.1002/jgh3.12490

**Published:** 2021-01-12

**Authors:** Naveen Anand, Akhilesh Sharma, Jimil Shah, Rakesh Kochhar, Shubh Mohan Singh

**Affiliations:** ^1^ Department of Psychiatry Postgraduate Institute of Medical Education and Research Chandigarh India; ^2^ Department of Gastroenterology Postgraduate Institute of Medical Education and Research Chandigarh India

**Keywords:** disability, Patient Health Questionnare‐9 score, World Health Organization Disability Assessment Schedule score, WHOQoL‐BREF score

## Abstract

**Background and Aim:**

Caustic ingestion is associated with long‐term sequelae in the form of esophageal and/or gastric cicatrization requiring endoscopic or surgical intervention. Quality of life (QoL) and disability in patients with caustic‐induced sequelae is less explored.

**Methods:**

In this prospective study, we included consecutive patients with symptomatic caustic‐induced esophageal stricture undergoing endoscopic dilatation. QoL was measured using the World Health Organization Quality of Life questionnaire (WHOQoL‐BREF). Disability was measured using the World Health Organization Disability Assessment Schedule 2.0 (WHODAS 2.0). Subjective dysphagia score was calculated by Likert scale.

**Results:**

A total of 42 patients were included in the study; 25 (59.5%) patients were male. Patients had poor WHOQoL‐BREF and WHODAS scores compared to normality data in all domains of the scores among both the genders. A majority (66.7%) of patients had a current psychiatric diagnosis, with the most common being mood disorder (50%) followed by suicidality (45.2%). Males had a higher prevalence of a previous psychiatric diagnosis compared to females, while females had a higher prevalence of suicidality. Dysphagia score had strong correlation with the WHOQoL (*r* = −0.66; *P* < 0.01) and WHODAS (*r* = 0.71; *P* < 0.01).

**Conclusion:**

Patients with esophageal stricture due to caustic ingestion on long‐term endoscopic dilatation have poor QoL, high prevalence of psychological morbidity, and disability.

## Graphical abstract

Caustic ingestion is associated with long‐term sequelae in the form of esophageal and/or gastric cicatrization requiring endoscopic or surgical intervention. We assessed quality of life, disability, and depression in a cohort of patients who ingested a caustic agent. These patients were found to have high levels of disability and depressive symptoms and poor quality of life.

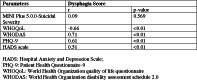

## Introduction

Ingestion of caustic substances is a long‐neglected major public health problem in many parts of the world.^1^, ^2^ Studies from western countries have shown a higher prevalence of accidental alkaline injuries, with 80% occurrence in the pediatric population.^3^, ^4^ In Asian countries, however, acidic injuries with suicidal intention predominate due to easy availability of over‐the‐counter acidic agents (like toilet cleaners).^1^, ^2^, ^5^ Acute caustic ingestion is associated with immediate complications, including vomiting, hematemesis, aspiration pneumonia, and—rarely—perforation. Late complications include esophageal stricture formation, gastric cicatrization, and—rarely—esophageal carcinoma.^6^, ^7^, ^8^

Long‐term consequences of caustic ingestion in the form of esophageal or gastric cicatrization may require multiple hospital visits, endoscopic treatment sessions, and sometimes surgical intervention, leading to high psychosocial morbidity.^9^ Although the physical effects and consequences of caustic ingestion have been explored in detail, the psychological effects in patients with sequelae of caustic ingestion have been studied less often. In some patients who require reconstructive surgery, failure to adequately manage the underlying psychiatric disorders is associated with poor functional outcomes and repeated suicide attempts.^10^ In addition, emergency surgery and pharyngeal reconstruction were shown to have a negative impact on quality of life (QoL) in patients with caustic injury.^9^, ^11^ However, there is little data on the psychological impact in patients requiring repeated endoscopic dilatation. This study was conducted to assess QoL, psychiatric morbidity, and disability in patients with esophageal stricture following caustic ingestion being treated with endoscopic dilatation.

## Methods

This was a prospective, single‐center study conducted in a tertiary care center in North India between January 2017 and June 2018 after approval was obtained from the Institutional Ethics Committee (IEC No: NK/3297/MD/2952).

### *Inclusion and exclusion criteria*

All consecutive adult patients (>18 years of age) with symptomatic caustic‐induced esophageal stricture with or without gastric cicatrization undergoing endoscopic treatment in the department of gastroenterology were included after obtaining written informed consent from the patient. Patients who had history of caustic ingestion within last 6 months, who were unwilling to be included in the study, were not compos mentis or were too ill to participate in the study, and were otherwise intoxicated during the period of assessment were excluded from the study.

### *Endoscopic management*

Details regarding caustic ingestion, such as nature and volume of the caustic, symptoms after ingestion, and initial endoscopic grading of mucosal injury (according to Zargar classification^12^), were reviewed. Site and extent of narrowing were noted during upper gastrointestinal endoscopy (GIF Q160 with outer diameter of 8.6 mm; Olympus Corp., Tokyo, Japan). Endoscopic management of esophageal stricture or gastric outlet obstruction was carried out as per methods described earlier.^13^, ^14^, ^15^ Dilatation was performed every 3 weeks using balloon dilators (Controlled Radial Expansion, CRE balloons, Boston scientific Corp, Natwick, MA, USA). Patients who did not achieve the target diameter of 15 mm in five dilatation sessions were labeled as having refractory strictures and were given intralesional triamcinolone.^13^Those who achieved a diameter of 15 mm were followed up every 3 months. Patients who had recurrence of symptoms were offered either continuation of dilatation or surgery. The cohort in the present study included patients who opted for continuation of dilatation. Subjective dysphagia score was calculated at each visit on a patient‐ratio 10‐point Likert scale, where 1 indicated no dysphagia and 10 indicated absolute dysphagia.^16^

### *Quality of life and psychological evaluation*

All psychological assessments were carried out by a single person (Naveen Anand) in a local language (Hindi) at least 6 months after the index ingestion episode to ensure that the acute physical and emotional effects of caustic ingestion had abated. QoL was measured using the WHOQoL‐BREF.^17^, ^18^ The WHOQoL‐BREF measures QoL in the domains of physical and psychological health, social relationships, and the environment. The raw scores obtained were transformed to a 0–100 scale, with higher scores indicating better QoL. Disability was measured using WHODAS 2.0.^19^ The WHODAS 2.0 is a validated instrument used to measure health and disability across different settings. It covers disability in six domains of functioning, namely, cognition, mobility, self‐care, getting along, life activities, and participation. Psychological morbidity was assessed using the Mini International Neuropsychiatric Interview 5.0.0 (MINI) to generate lifetime and current psychiatric diagnoses,^20^ and the PTSD diagnostic scale for diagnostic and statistical manual of mental disorders (DSM) 5 (PTSD‐5) was used to generate a probable diagnosis of posttraumatic stress disorder (PTSD).^21^ We used the Patient Health Questionnare‐9 (PHQ‐9) to assess for depressive symptom severity.^22^ Suicidality was assessed using the ninth item on the PHQ‐9 questionnaire.^22^

### *Statistical analysis*

All data were analyzed using SPSS software.^23^ The various QoL scores and disability scores were calculated and correlated with dysphagia scores and duration of treatment (< or ≥1 year). Data were explored for any outliers, errors, and missing values. The data were checked for normal distribution using the Kolmogorov–Smirnov test. For normally distributed data, a student t test was used for continuous variables, while for skewed data, a Mann–Whitney *U* test was used. Dichotomous variables were compared using the Chi square test. Quantitative data were described as mean and SD with 95% confidence intervals. Categorical data were shown as proportions. Correlation studies were carried out using Pearson correlation between QoL, disability scores, and dysphagia scores. Pearson correlation coefficient value of 0–0.19 was regarded as very weak, 0.2–0.39 as weak, 0.40–0.59 as moderate, 0.6–0.79 as strong, and 0.8–1.0 as very strong correlation^24^ A *P* value of less than 0.05 was taken as statistically significant.

## Results

Of the 64 patients of caustic‐induced sequelae seen by us during the study period, 7 had isolated gastric outlet obstruction, 12 had a history of caustic ingestion <6‐month duration, and 3 did not give consent for study. A total of 42 patients fulfilled inclusion criteria during the study period. The mean age of included patients was 31.8 ± 13.4 years, and 25 (59.5%) patients were male. Thirty‐seven (88.1%) patients had a history of acid ingestion. All patients had esophageal involvement, while 12 patients also had gastric involvement. A total of 22 patients (52.4%) had a history of caustic ingestion more than 12 months previously, while 20 patients (47.6%) had history of caustic ingestion within the last 12 months (Table 1).

**Table 1:** Sociodemographic profile of study population

Parameters	*n* = 42	
Age (years)	31.76 ± 13.37	
Males	25 (59.5%)	
Nature of caustic ingestion		
Acid	37 (88.1%)	
Alkali	5 (11.9%)	
Modality of caustic ingestion		
Accidental (male/female)	20/7	*P* = 0.01
Suicidal (male/female)	5/10	
Site of involvement		
Esophageal	42 (100.0%)	
Gastric	12 (28.6%)	
Duration from caustic ingestion		
<12 months	22 (52.4%)	
>12 months	20 (47.6%)	

### *Quality of life and disability in survivors of corrosive ingestion*

QoL among patients with caustic‐induced sequelae was lower compared to normality in all domains of the WHOQoL score^17^, ^25^, ^26^ (Table 2). Mean scores in physical, psychological, social, and environmental domains were 55.8 ± 14.7, 55.8 ± 16.3, 56.9 ± 24.1, and 52.9 ± 26.3, respectively, which were lower compared to normality.^17^, ^25^, ^26^ These scores in different domains were similar among both the genders (Table 2). Similarly, mean disability scores in certain domains of WHODAS score were higher compared to normality.^19^, ^27^, ^28^ The mean disability scores of getting around, self‐care, life activities, and participation in society were 29.6 ± 25.9, 24.1 ± 21.1, 38.3 ± 31.4, and 53.6 ± 27.2, respectively, which indicated higher disability scores compared to normality. However, both the genders had similar disability in various parameters, including life activities and social participation, after caustic sequelae (Table 3).

**Table 2:** Quality of life in patients of corrosive ingestion using WHOQoL score

Domain	Male (*n* = 25)	Female (*n* = 17)	*P*‐value
Domain 1‐Physical	53.86 ± 15.29	58.61 ± 13.6	0.31
Domain 2‐Psychological	55.83 ± 15.68	55.63 ± 17.61	0.97
Domain 3‐Social relations	58.67 ± 23.26	54.41 ± 25.87	0.58
Domain 4‐Environment	52.25 ± 26.74	53.86 ± 26.44	0.85
WHOQOL	53.65 ± 18.78	55.15 ± 18.98	0.80

WHOQoL, World Health Organization Quality of Life Questionnaire.

**Table 3:** Disability in patients of caustic ingestion using WHODAS score

Domain	Male (*n* = 25)	Female (*n* = 17)	*P*‐value
Understanding and communication	18.67 ± 21.65	17.93 ± 17.35	0.91
Getting around	31.2 ± 25.09	27.35 ± 27.79	0.64
Self‐care	26.0 ± 23.57	23.57 ± 27.79	0.49
Getting along with people	18.40 ± 18.30	20.29 ± 24.90	0.78
Life activities	40.0 ± 31.18	55.88 ± 26.45	0.68
Participation in the society	32.89 ± 21.96	50.37 ± 28.81	0.53
Overall disability	32.89 ± 21.96	28.85 ± 21.02	0.55

WHODAS, World Health Organization Disability Assessment Schedule 2.0.

### *Psychiatric morbidity*

In our study, 36 (85.7%) patients had lifetime psychiatric diagnosis, and 28 (66.7%) patients had current psychiatric diagnosis (Table 4). The most common current psychiatric diagnosis was mood disorder (50%) followed by suicidality (45.2%), anxiety disorder (35.7%), and substance abuse (26.2%). Males had a higher frequency of previous psychiatric diagnosis compared to females as per MINI 5.0.0 score (96 *vs* 70.6%; *P* = 0.02). Prevalence and type of mood disorder and anxiety disorder was similar among males and females. Prevalence of alcohol dependence was higher in males compared to females (24 *vs* 0%; *P* = 0.03). Severity of suicidality was higher in females compared to males, with a higher prevalence of moderate to severe suicidality (*P* = 0.004) and a higher risk of current suicidality (5.4 ± 6.5 *vs* 1.0 ± 1.8; *P* < 0.01). Values of posttraumatic stress disorder diagnostic scale, hospital anxiety and depression scale, PHQ‐9 scales were equal among males and females (Table 4).

**Table 4:** Psychiatric morbidity in patients with caustic ingestion

Parameters	Male (*n* = 25)	Female (*n* = 17)	*P*‐value
Previous psychiatric diagnosis	24 (96.0%)	12 (70.6%)	0.02
Current psychiatric diagnosis	17 (68.0%)	11 (64.7%)	0.82
Mood disorder	14 (56.0%)	7 (41.2%)	0.35
Major depressive episode, current	4 (16.0%)	3 (17.6%)	0.88
Major depressive episode, past	5 (20.0%)	3 (17.6%)	0.85
Major depressive episode, recurrent	2 (8.0%)	1 (5.9%)	0.79
Dysthymia, past	4 (16.0%)	2 (11.8%)	0.70
Anxiety disorder	11 (44.0%)	4 (23.5%)	0.17
Panic disorder	3 (12%)	4 (23.5%)	0.33
Mixed anxiety depression	2 (8.0%)	0 (0.0%)	0.23
Specific phobia	1 (4.0%)	0 (0.0%)	0.40
Posttraumatic stress disorder	1 (4.0%)	0 (0.0%)	0.40
Generalized anxiety disorder	2 (8.0%)	0 (0.0%)	0.23
Adjustment disorder	2 (8.0%)	0 (0.0%)	0.23
Substance use disorder	11 (44.0%)	0 (0.0%)	0.23
Alcohol dependence	6 (24.0%)	0 (0.0%)	**0.03**
Alcohol abuse	3 (12.0%)	0 (0.0%)	0.14
Other substance dependence	7 (28.0%)	0 (0.0%)	**0.02**
Suicidality	10 (40.0%)	9 (52.9%)	0.41
Low	10 (40.0%)	4 (23.5%)	**0.004**
Moderate	0 (0.0%)	4 (23.5%)	
High	0 (0.0%)	1 (5.9%)	
Risk of current suicidality	1.04 ± 1.77	5.35 ± 6.45	**<0.01**
PDS‐5 total score	21.04 ± 12.08	21.88 ± 13.35	0.83
Symptom severity	15.6 ± 10.36	16.24 ± 10.89	0.85
Distress and interference	2.68 ± 1.6	3.18 ± 2.13	0.39
HADS total score	10.04 ± 9.7	11.59 ± 9.12	0.61
Anxiety subscale	5.96 ± 5.01	6.35 ± 4.82	0.80
Depression subscale	4.08 ± 4.96	5.24 ± 4.56	0.44
PHQ‐9 total score	6.16 ± 6.18	7.82 ± 6.61	0.41

Values given in bold are *p* values that were found significant.

HADS, Hospital Anxiety and Depression Scale; PDS‐5, Posttraumatic stress disorder diagnostic scale for DSM 5; PHQ‐9, Patient Health Questionnaire‐9.

### *Comparison of various scores with mood disorder, anxiety disorder, suicidality, duration of illness, and dysphagia score*

Patients with a mood disorder and suicidality had poor WHOQoL scores, WHODAS score, PHQ‐9 score, and HADS scale compared to patients without a mood disorder. However the scores of these variables did not differ significantly on appropriate statistical tests when compared in patients with and without anxiety disorder and in patients with various durations of illness (Table 5). Dysphagia score had a strong correlation with the WHOQoL (*r* = −0.66; *P* < 0.01), WHODAS (*r* = 0.71; *P* < 0.01), and PHQ‐9 (*r* = 0.61; *P* < 0.01) scores (Table 6).

**Table 5:** Comparison of mood disorder, anxiety disorder, suicidality, and duration of illness with various parameters

	Mood disorder			Anxiety			Suicidality			Duration of illness		
Parameters	With	Without	*P*‐value	With	Without	*P*‐value	With	Without	*P*‐value	<1 year	>1 year	*P*‐value
WHOQoL	69.90 ± 19.15	83.62 ± 14.27	**0.01**	71.67 ± 15.51	79.59 ± 19.01	0.17	68.47 ± 16.16	83.61 ± 16.91	**<0.01**	74.14 ± 15.96	79.38 ± 19.99	0.35
WHODAS	41.57 ± 25.31	25.39 ± 18.51	**0.02**	35.37 ± 21.21	32.43 ± 24.83	0.70	43.31 ± 22.98	25.36 ± 20.84	**0.01**	34.06 ± 20.98	32.9 ± 26.07	0.87
PHQ‐9	9.0 ± 7.45	4.67 ± 4.09	**0.02**	7.93 ± 6.87	6.22 ± 6.05	0.41	9.47 ± 6.91	4.65 ± 4.95	**0.01**	7.19 ± 5.09	6.48 ± 7.47	0.75
HADS scale	14.71 ± 10.24	6.62 ± 6.43	**<0.01**	12.33 ± 9.6	9.74 ± 9.32	0.39	15.11 ± 10.34	7.0 ± 6.76	**<0.01**	11.14 ± 7.98	10.19 ± 10.79	0.72
PTSD symptom severity	21.43 ± 8.34	10.29 ± 9.45	**<0.01**	15.07 ± 9.71	16.3 ± 10.99	0.72	18.74 ± 9.37	13.48 ± 10.89	0.11	16.57 ± 9.08	15.14 ± 11.85	0.66

Values given in bold are *p* values that were found significant.

HADS, Hospital Anxiety and Depression Scale; PHQ‐9, Patient Health Questionnaire‐9; WHODAS, World Health Organization disability assessment schedule 2.0; WHOQoL, World Health Organization quality of life questionnaire.

**Table 6:** Correlation of dysphagia score with various parameters

	Dysphagia Score	
Parameters	*r*	*P*‐value
MINI Plus 5.0.0‐Suicidal Severity	0.09	0.569
WHOQoL	−0.66	<0.01
WHODAS	0.71	<0.01
PHQ‐9	0.61	<0.01
HADS scale	0.51	<0.01

HADS, Hospital Anxiety and Depression Scale; PHQ‐9, Patient Health Questionnaire‐9; WHODAS: World Health Organization disability assessment schedule 2.0; WHOQoL, World Health Organization quality of life questionnaire.

There was no statistical difference in the study variables with respect to the duration of illness.

## Discussion

In this prospective study, we evaluated QoL, disability, and psychological morbidity in patients with caustic‐induced esophageal stricture requiring repeated endoscopic dilatation. We observed that patients had poor QoL and more disability compared to normal data. Males had a higher prevalence of previous psychiatric diagnosis and alcohol dependence compared to females, while females had a higher prevalence of moderate to severe suicidality.

A majority of our patients had presented with accidental acid ingestion. This has been observed in nearly all the studies from India.^1^, ^2^, ^5^ The likely explanation for this is easy availability of over‐the‐counter acids (toilet cleaners) in India.^1^, ^2^, ^5^ Alcohol dependence or abuse was more common in males, which might be the reason for higher accidental caustic ingestion in an inebriated state in males. Female patients had a higher prevalence of suicidality and risk of current suicidality, which explains the higher incidence of caustic ingestion with suicidal intention in females. However, this self‐reporting of intention of caustic ingestion could also be biased due to associated social stigmatization and legal implications.

There are only a few studies in the literature on QoL in patients with caustic ingestion, and they have addressed different issues using different instruments.^9^, ^10^, ^11^, ^29^, ^30^ Faron *et al*. had evaluated QoL in patients with survivors of a caustic ingestion using EORTC QLQ‐OG25 and SF12 scores. In their study, the majority (99 of 134 [74%]) of patients had undergone some form of surgical interventions due to caustic‐induced complications. Patients had poor QoL scores compared to normal individuals in both physical and mental domains.^9^ Raynaud *et al*. also evaluated QoL in patients with caustic ingestion who needed emergency surgery using the EORTC QLQ‐OG25 score. Patients who could be managed with conservative management performed better with better QoL scores compared to patients who required emergency surgery.^11^

In our study, we used the WHOQoL‐BREF score to measure the QoL in patients with caustic sequelae, which is a validated and recommended score for the assessment of QoL. Although we had not used controls, patients with caustic‐induced sequelae had poor QoL in all domains of the score (physical, psychological, social, and environmental) compared to previously described values for normal individuals.^17^, ^25^, ^26^ Moreover, the dysphagia score strongly correlated with the WHOQoL score, suggesting that a higher dysphagia score was associated with poorer QoL. Such impairment of QoL in patients with caustic sequelae was comparable to impaired QoL in patients with other disorders such as neurodegenerative disease, stroke, and Crohn's disease and was worse than disorders such as schizophrenia, mild dementia, and depression.^31^

We used the WHODAS 2.0 score to evaluate disability in our cohort. Similar to QoL, patients had more disability in certain domains (participation in society, life activity, getting around, and self‐care) when compared to previously described values for normal individuals.^19^, ^27^, ^28^ Dysphagia scores strongly correlated with WHODAS scores, suggesting that a higher dysphagia score is associated with more disability. Similar findings have also been noted for mental illnesses such as bipolar disorder.^32^ Argyriou *et al*. evaluated disability in 200 patients with inflammatory bowel disease using the WHODAS 2.0 score. In their study as well, patients had more disability in all domains of score (mobility, self‐care, relationships, life activities, and social participation) compared to normal individuals.^33^

In our study, almost two‐thirds of our patients had a current psychiatric morbidity, with mood disorder (50%) and suicidality (45.2%) being the most common. Males were found to have a higher frequency of substance abuse, while females were found to have a higher risk of suicidality, especially moderate and higher risk of suicidality. Earlier studies have also found higher prevalence of psychiatric morbidity in patients with caustic ingestion, ranging from 25 to 86%.^10^, ^29^, ^34^ In those studies as well, females were found to have a higher risk of suicidality compared to males.^29^ Moreover, patients with a mood disorder or suicidality had poor QoL, higher disability scores, and depressive scores compared to patients without a mood disorder or suicidality. Survivors of caustic ingestion need long‐term psychosocial follow‐up and care rather than purely symptomatic treatment for better outcomes. Moreover, it is also probable that the management of psychological distress in people with physical disorders leads to better outcomes with regard to a variety of parameters.^35^ While our study was not designed to assess for causality, we found that disability, psychiatric morbidity, QOL, and symptomatic severity were strongly correlated with each other. The variables included in the study did not statistically differ with respect to the duration of illness. Thus, the deficits seem to be stable and indicate a continuing need for psychosocial and material assistance.

In our study, we included a stable cohort of patients for a representative population with long‐term sequelae of caustic ingestion. We evaluated QoL, disability, and psychological morbidity using well‐validated instruments in our cohort, which is the first of its kind. However, our study included only those patients who were on long‐term dilatation because of mostly refractory stricture. There are indeed patients with caustic‐induced esophageal stricture who require fewer dilatations, and their QoL and disability may not be as worse as in this study population. The limitations include the cross‐sectional nature of the assessment and the lack of a control group. We also did not conduct any formal assessments of personality of the participants, which may have had a bearing on the index episode of caustic ingestion and the reaction thereof. Moreover, included patients were undergoing repeated endoscopic dilatation sessions, which itself add psychological morbidity and confound the psychosocial evaluation.

To conclude, our study shows that patients with esophageal stricture due to caustic ingestion on long‐term endoscopic dilatation have high prevalence of underlying psychological morbidity, impaired QoL, and higher disability. Dysphagia score strongly correlates with QoL score and disability score, with higher dysphagia being associated with poorer scores.
